# Longitudinal analysis of viral dynamics in HIV^+^-to-HIV^+^ HOPE Act kidney-transplant recipients

**DOI:** 10.1172/JCI181560

**Published:** 2024-09-10

**Authors:** Tatianna Travieso, Hannah Stadtler, Naseem Alavian, Feng Gao, Mary Klotman, Cameron Robert Wolfe, Maria Blasi

**Affiliations:** 1 Department of Medicine, Division of Infectious Diseases and; 2 Duke Human Vaccine Institute, Duke University School of Medicine, Durham, North Carolina, USA.; 3 Institute of Molecular and Medical Virology, School of Medicine, Jinan University, Guangzhou, Guangdong Province, China.

**Keywords:** AIDS/HIV, Transplantation, Chronic kidney disease, Organ transplantation

## Abstract

**BACKGROUND:**

The HIV Organ Policy Equity (HOPE) Act allows individuals living with HIV to accept organs from donors with HIV. This practice widens the pool of available organs, but also presents important virological issues, including the potential for HIV superinfection of the recipient, viral persistence in the kidney, and loss of virological control.

**METHODS:**

We addressed these issues by performing in-depth longitudinal viral sequence analyses on urine, blood, and urine-derived renal epithelial cells from 12 recipients of HIV^+^ kidney allografts.

**RESULTS:**

We amplified donor-derived HIV-1 env sequences in 5 out of 12 recipients after transplant. These donor-derived env sequences were amplified from recipient urine, urine-derived renal epithelial cells, and plasma between 12 and 96 hours after transplant and remained detectable up to 16 days after transplant. Env sequences were also detected in kidney biopsies taken from the allografts before implantation in 6 out of the 12 transplant cases, indicating the presence of donor virus within the organ. One recipient had a viremic episode 3.5 years after transplantation as a result of antiretroviral therapy (ART) interruption. Only recipient strain viral sequences were detected in blood, suggesting that the donor virus, if still present, was not reactivated during the temporary ART withdrawal.

**CONCLUSIONS:**

This study demonstrates that the HIV env sequences in a donor kidney can be amplified from biopsies taken from the allograft before implantation and can be detected transiently in blood and urine samples collected from the organ recipients after transplantation.

**FUNDING:**

National Institute of Diabetes and Digestive and Kidney Diseases (NIDDK) grant number R01DK131497.

## Abstract 2

HIV sequences in donors’ kidneys can be amplified from the allografts’ biopsies and be detected transiently in the recipients’ blood and urine.

## Graphical abstract

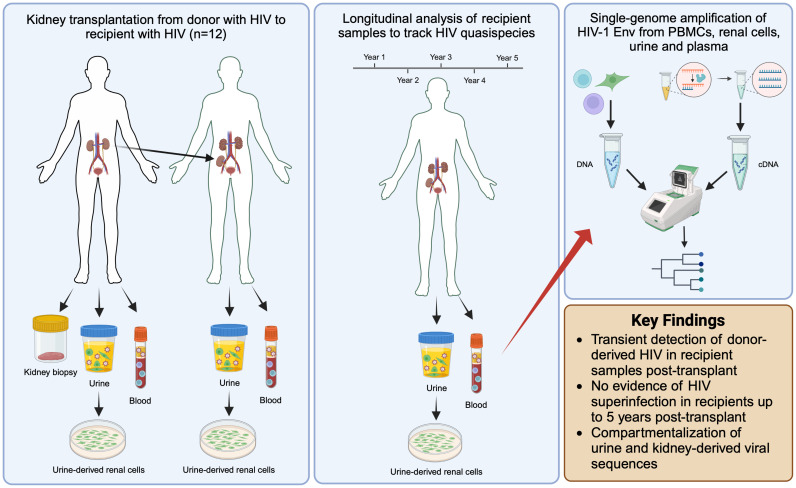

## Introduction

People living with HIV (PLWH) are at high risk of end-stage renal disease (ESRD) due to direct kidney injury caused by HIV, associated comorbidities including hypertension and diabetes mellitus, coinfections, and renal toxicity from antiretroviral therapy (ART). On dialysis, PLWH fare far worse than HIV-negative individuals, with 5-year survival rates of 62.7% as compared with 94.4% (1). Given the challenges and increased mortality associated with dialysis in these patients, kidney transplantation provides a better long-term solution to ESRD in PLWH (2). The worldwide organ shortage, which was only exacerbated by the COVID-19 pandemic (3), has limited the implementation of this treatment. As of 2013, with the passage of the HIV Organ Policy Equity (HOPE) Act, the donation of HIV-positive organs for recipients with HIV has widened the pool of available organs for PLWH and ESRD (4). Although a newer practice within the US, organ transplants between donors and recipients infected with HIV have been performed in South Africa since 2008 (5). The first analysis of 27 HIV-positive–to–HIV-positive kidney transplants performed in South Africa demonstrated acceptable patient and graft survival at 5 years (74% and 84%, respectively) (6). Key differences between the pool of deceased donors with HIV in South Africa and in the United States, including the increased prevalence of ART-resistant strains in ART-naive patients within the US (7), present important unanswered virological questions surrounding HIV-positive–to–HIV-positive solid organ transplantation. One issue is the potential introduction of an ART-resistant virus leading to superinfection of the recipient and potential recombination between donor and recipient viruses. HIV superinfection has been reported with all modes of HIV transmission, including sexual transmission and intravenous drug use (8). Although effective ART should reduce the risk of superinfection and preliminary findings have not provided evidence of donor-derived superinfection (9–11), the impact of immune suppression, the challenges of managing immunosuppressive and antiretroviral drugs (12), and the high viral load (VL) of some deceased donors with HIV could increase that risk. Another concern is the amount of virus present in the transplanted kidneys; the transplanted organs may contain infected PBMCs, cell-free virus, infected interstitial inflammatory cells, and infected renal epithelial cells (13, 14), and the viral populations present in these compartments are genetically different from those found in blood (15, 16). Indeed, collective work from our lab has demonstrated that HIV-infected renal tubular epithelial (RTE) cells can produce infectious viruses in vitro (17) and that the kidney represents a compartment for HIV replication separate from blood in vivo (16). Additionally, a study by Canaud et al. demonstrated that up to 68% of recipients with HIV receiving kidneys from HIV-negative donors had HIV infection of allograft renal epithelial cells after transplantation, despite the absence of detectable plasma viremia during the posttransplant period (18). HIV infection of kidney cells is a concern, as it could affect long-term allograft survival (19, 20). In the South African study that assessed the clinical outcome of HIV-positive–to–HIV-positive kidney transplantation, 3 of 27 subjects developed recurrent HIV-associated nephropathy (HIVAN) (6), consistent with HIV infection of the kidney (21). We have previously developed a noninvasive approach for studying the viral dynamics in the urinary tract and demonstrated that HIV envelope glycoprotein (*env*) gene sequences can be amplified from urine supernatants of PLWH with detectable viremia and that those sequences reveal the presence of a unique compartment separate from blood (15). In our initial virological analysis of the first HOPE Act kidney transplant performed in our center, we showed that donor-derived viral sequences could be identified in the biopsy of the kidney allograft as well as in blood, urine, and urine-derived renal epithelial cells of the transplant recipient up to 16 days after transplant (22), indicating the need for longitudinal monitoring of these transplant cases. In the current study, we monitored 12 HOPE Act kidney transplant recipients up to 5 years after transplant to assess (a) the frequency and duration of detection of viral quasispecies from the donors with HIV in urine and blood of the recipients with HIV following transplantation, (b) the presence of HIV in kidney biopsy of the donor organ as well as within renal epithelial cells isolated from the urine of the recipients, and (c) the viral dynamics in the recipients over time.

## Results

### Participant characteristics.

Twelve HIV-positive–to–HIV-positive kidney transplant recipients were monitored for up to 5 years after transplantation. All the recipients were ART compliant at the time of transplant, with VLs below 20 copies/mL (Table 1). Four of the 12 recipients had a history of opportunistic infection. HIV VL remained suppressed or low for all recipients throughout the posttransplantation follow-up period except for 1 patient who had a viremic episode (VL of 20,000 copies per mL) 3.5 years after transplantation as a result of ART interruption. Among the 12 donors with HIV, 8 were reported to be on ART (Table 1) and 6 of these donors had suppressed viremia (<20 copies/mL). Three of the 4 untreated donors were viremic, with a VL ranging from 15,244 copies per mL to 183,236 copies per mL (Table 1). The fourth untreated donor was thought to have had acute HIV infection, as their Ultrio Elite HIV-1/2 nucleic acid amplification test (NAAT) was reactive, but an HIV enzyme immunoassay to groups M and O antibody was unreactive. VL was not tested during organ procurement for this donor with acute HIV infection; however, we obtained a small aliquot of plasma to perform single genome amplification and sequence analysis.

### Identification of donor virus within recipient samples is largely dependent on donor viremia.

Of the 6 transplant cases involving donors with nonsuppressed viremia, 4 resulted in identification of donor-derived sequences in the recipient’s samples after transplant (HOPE 1, 4, 14, and 15). Of the 6 remaining transplant cases (all receiving an allograft from donors with an undetected VL), only 1 (HOPE 11) resulted in the identification of donor-derived sequences in the recipient’s samples. Additionally, we amplified HIV *env* sequences in 6 out of the 12 kidney biopsies taken from the allografts before implantation (HOPE 1, 5, 6, 8, 9, and 11 donors).

**Figure 1:**
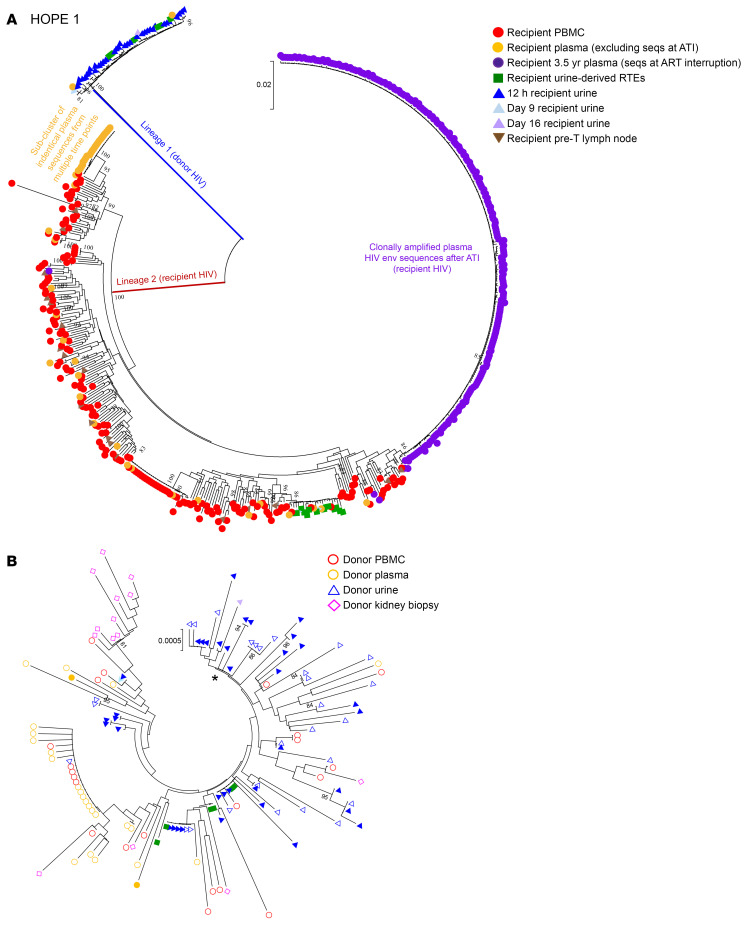
Phylogenetic tree analysis of HIV *env* sequences amplified from Hope 1 recipient before and up to 5 years after kidney transplant. (**A**) Neighbor-joining phylogenetic tree that includes all of the HIV envelope sequences amplified from blood, urine, and LN samples obtained from the kidney-transplant recipient with HIV before and up to 5 years after transplantation of a kidney from a donor with HIV. Two separate viral lineages (lineages 1 and 2) were identified in the recipient up to 16 days after transplantation. Bootstrap values over 80% are indicated. (**B**) All the HIV quasispecies in lineage 1 that were amplified from the recipient’s urine (solid blue triangles for cell-free viral RNA and green squares for viral DNA associated with RTE cells) and plasma (solid orange circles) between 12 hours and 16 days after transplantation are genetically related to the donor virus (open shapes) and genetically distant from the viral sequences amplified from the recipient’s PBMCs, plasma, LNs, and urine-derived RTE cells before transplantation. Several HIV env sequences were also amplified from the kidney biopsy taken from the allograft before implantation (open pink diamonds), the majority of which were compartmentalized from the rest of samples analyzed (*P* value of 0.016 using a codon-based test of positive selection). The asterisk demarks a group of HIV envelope sequences amplified from either donor urine or recipient urine collected after transplantation that clustered separately from blood-derived sequences.

We had previously reported (22) that donor-derived viral sequences were detected up to 16 days after transplant in the HOPE 1 recipient’s samples (Supplemental Table 1; supplemental material available online with this article; https://doi.org/10.1172/JCI181560DS1). In this recipient, a total of 100 HIV-1 env sequences were amplified between 12 hours to 16 days after transplant from urine, plasma, urine-derived renal epithelial cells, PBMCs, and nonadherent urinary cells. Phylogenetic analysis demonstrated that 47 of those *env* sequences belonged to a separate viral lineage (lineage 1), not previously detected in samples collected from the recipient before transplantation (Figure 1A). To confirm that the sequences detected in recipient’s urine, plasma, and urine-derived renal epithelial cells were genetically related to the donor virus, we included amplified sequences from donor samples in the phylogenetic analysis, all of which aligned with the lineage 1 sequences from the recipient, indicating genetic relation (Figure 1B). Additionally, some of the urine-derived sequences, regardless of the source (donor urine or recipient urine collected after transplantation), formed a separate cluster from the blood sequences (Figure 1B), suggesting that those urine viruses were primarily produced by infected cells intrinsic to the transplanted kidney, rather than originating from donor’s PBMCs or cell-free plasma viruses. A total of 20 *env* sequences (4 from urine collected before transplantation and 16 after transplant) were amplified from the cultured urine-derived renal epithelial cells (Supplemental Table 1), 14 of which corresponded to the recipient viruses (green squares in lineage 1, Figure 1A) and 6 to the donor viruses (green squares in lineage 2, Figure 1, A and B). Interestingly, we observed that those 6 donor *env* sequences were closely related to the *env* sequences amplified from cell-free urine of the recipient at 12 hours after transplantation (solid blue triangles in Figure 1A), supporting kidney epithelial cells as a source of cell-free donor-derived viruses in urine. Several HIV env sequences were also amplified from the kidney biopsy taken from the allograft before implantation, the majority of which were compartmentalized from the rest of samples analyzed (Figure 1B). All the urine (69/69), renal epithelial cells (20/20), and kidney biopsy sequences (14/14) were predicted to use CCR5 coreceptors (CCR5 false-positive rate < 10%). Follow-up analysis of blood and urine samples collected from this recipient up to 5 years after transplantation failed to detect any donor virus, even during a viremic episode 3.5 years after transplantation as a result of ART treatment interruption. Interestingly, phylogenetic analysis of the 245 sequences amplified from plasma during ART interruption demonstrated the presence of clusters of identical and nearly identical HIV *env* sequences (Figure 1A and Supplemental Figure 1), suggesting reactivation of a clonally expanded and transcriptionally active viral reservoir (23).

**Figure 2:**
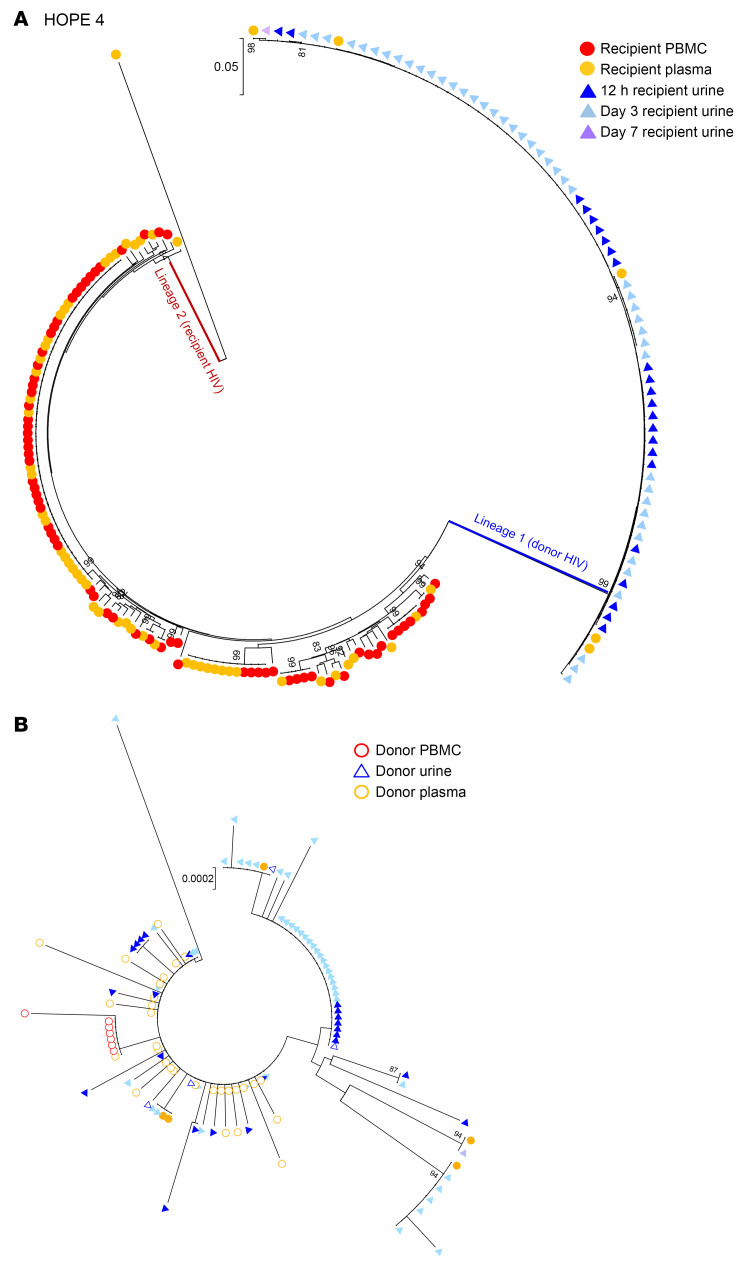
Phylogenetic tree analysis of HIV *env* sequences amplified from Hope 4 recipient before and up to 3.25 years after kidney transplant. (**A**) Neighbor-joining phylogenetic tree that includes all of the HIV envelope sequences amplified from blood and urine samples obtained from the kidney-transplant recipient with HIV before and up to 3.25 years after transplantation of a kidney from a donor with HIV. Two separate viral lineages (lineages 1 and 2) were identified in the recipient up to 7 days after transplantation. Bootstrap values over 80% are indicated. (**B**) All the HIV quasispecies in lineage 1 that were amplified from the recipient’s urine (solid blue triangles for cell-free viral RNA) and plasma (solid orange circles) between 12 hours and 7 days after transplantation are genetically related to the donor virus (open shapes) and genetically distant from the viral sequences amplified from the recipient’s PBMCs and plasma before transplantation. All the urine sequences (73/73) were predicted to use CCR5 coreceptors (CCR5 false-positive rate <10%).

For HOPE 4, the second of the transplant cases where donor-derived sequences were identified in the recipient’s samples, a total of 107 HIV-1 *env* sequences were amplified from the recipient’s urine, plasma, and PBMCs from 12 hours to 7 days after transplant. Seventy-four of those sequences (69%) were donor derived and were detected in both urine and plasma samples (Supplemental Table 1). As with HOPE 1, phylogenetic analysis of the *env* sequences amplified from the HOPE 4 recipient’s urine and blood (both cell-free and cell-associated viruses) collected before (pre-T) and up to 7 days after transplant showed 2 distinct viral lineages (Figure 2A). Lineage 1 included all the *env* sequences amplified from the urine collected 12 hours, 3 days, and 7 days after transplant. All of these lineage 1 sequences were genetically related to the donor’s HIV strain (Figure 2B). The second lineage included all the *env* sequences amplified from samples collected before and after transplantation that belonged to the recipient HIV strain. In line with our previous reports (15, 22, 24), the majority of the HIV *env* sequences amplified from urine clustered together and several of them were identical to each other. Additionally, all the donor-derived sequences amplified from the recipient’s plasma after transplantation were identical to urine-derived sequences (Figure 2B), suggesting a common source. Follow-up analysis of blood and urine samples collected from this recipient up to 3.25 years after transplantation failed to detect any donor virus.

**Figure 3:**
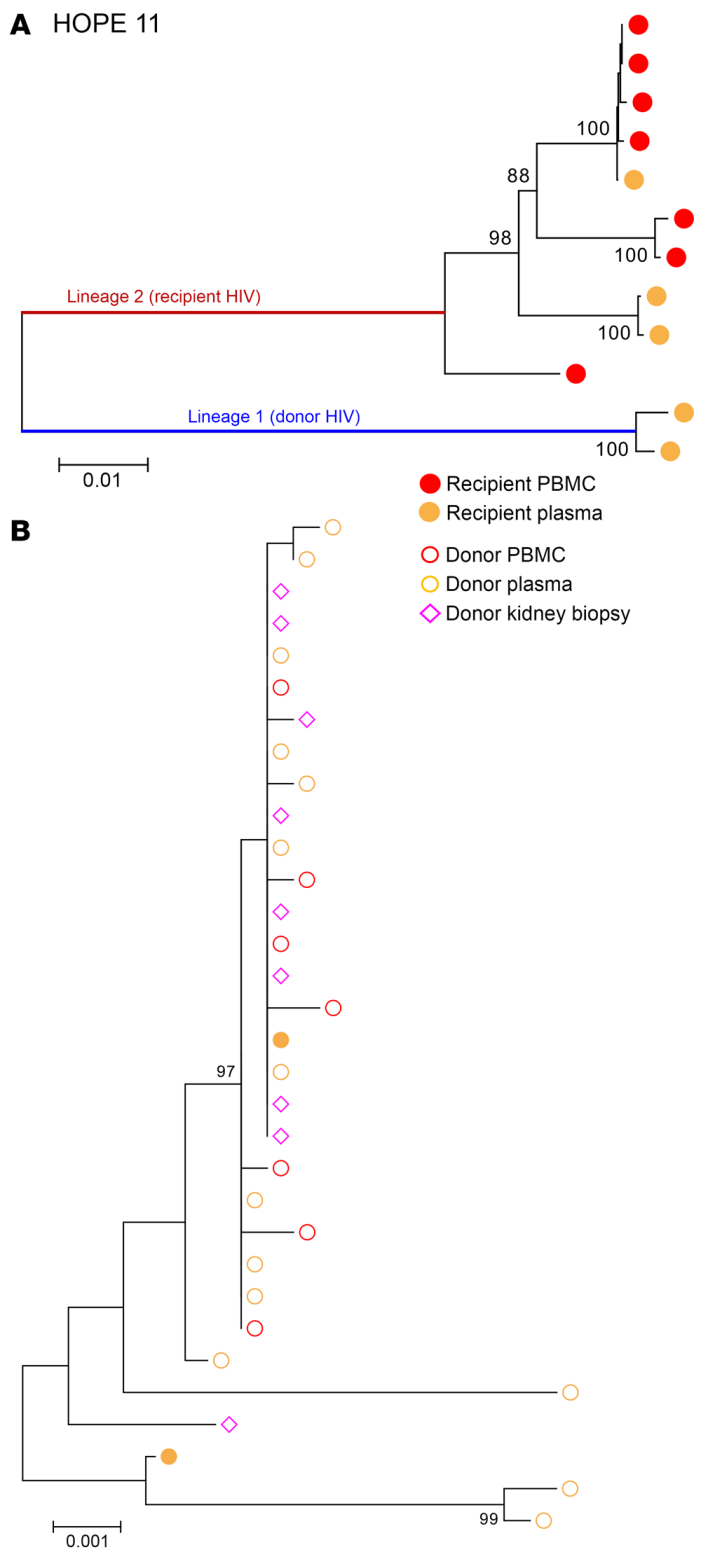
Phylogenetic tree analysis of HIV *env* sequences amplified from Hope 11 recipient before and up to 9 months after kidney transplant. (**A**) Neighbor-joining phylogenetic tree that includes all of the HIV envelope sequences amplified from blood and urine samples obtained from the kidney-transplant recipient with HIV before and up to 9 months after transplantation of a kidney from a donor with HIV. Donor virus was detected in this recipient’s plasma 24 hours after transplantation. (**B**) Two HIV env sequences in lineage 1 that were amplified from the recipient’s plasma (solid orange circles) at 24 hours after transplantation are genetically related to the donor virus (open shapes). Additionally, several HIV env sequences were amplified from the kidney biopsy (open pink diamonds) taken from the allograft before implantation. Bootstrap values over 80% are indicated. All the donor- and recipient-derived sequences were predicted to use CCR5 coreceptors (CCR5 false-positive rate <10%).

HOPE 11 was the only transplant case in which we detected donor-derived HIV-1 *env* sequences in a recipient receiving an allograft from a donor with an undetectable VL (<20 copies/mL). These donor-derived viral sequences were only detected in plasma 24 hours after transplant (Supplemental Table 1) and accounted for 50% of the sequences detected at that time point (2/4) (Figure 3A). Though we detected several HIV *env* sequences in the donor kidney biopsy, they did not show the same degree of compartmentalization as the HIV env sequences amplified from the kidney biopsy of the HOPE 1 donor, and the 2 donor-derived plasma sequences detected 24 hours after transplant were similarly interspersed among donor plasma, PBMC, and kidney biopsy sequences (Figure 3B). Follow-up analysis of blood and urine samples collected from this recipient up to 9 months after transplantation failed to detect any donor virus.

**Figure 4:**
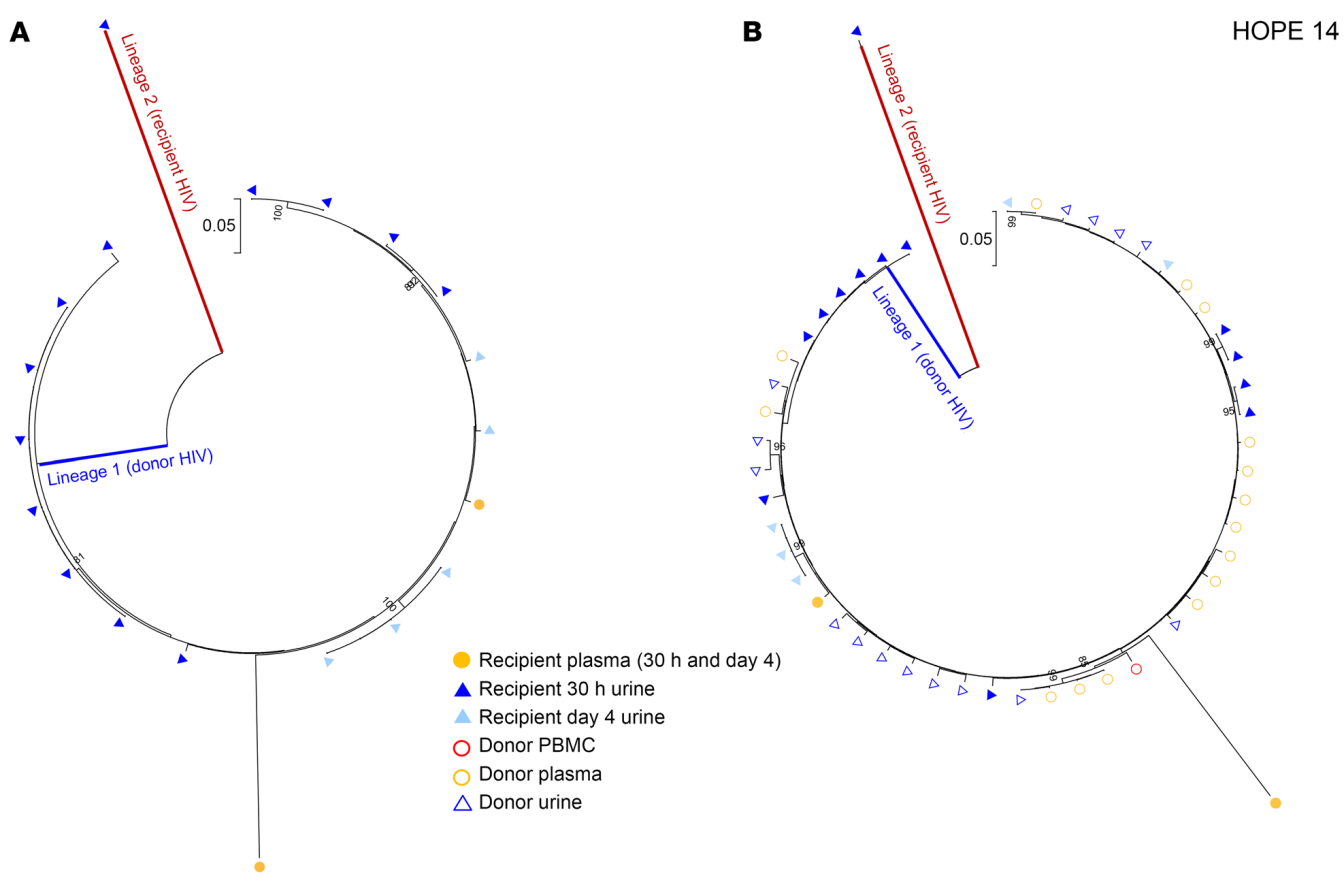
Phylogenetic tree analysis of HIV *env* sequences amplified from Hope 14 recipient before and up to 1 month after kidney transplant. (**A**) Neighbor-joining phylogenetic tree that includes all of the HIV envelope sequences amplified from blood and urine samples obtained from the kidney-transplant recipient with HIV before and up to 1 month after transplantation of a kidney from a donor with HIV. Two separate viral lineages (lineages 1 and 2) were identified in the recipient up to 4 days after transplantation in both urine and plasma. Bootstrap values over 80% are indicated. (**B**) All the HIV quasispecies in lineage 1 that were amplified from the recipient’s urine (solid blue triangles for cell-free viral RNA) and plasma (solid orange circles) between 30 hours and 4 days after transplantation are genetically related to the donor virus (open shapes). No HIV env sequences could be amplified from urine and blood samples collected from the recipient before transplantation. All the donor-derived HIV sequences were predicted to use CCR5 coreceptors, while the urine-derived HIV sequence shown in lineage 2 (recipient HIV) was predicted to use CXCR4 (CCR5 false-positive rate <10%),

HOPE 14 presents a case where almost 100% (19/20) of the HIV *env* sequences amplified in the recipient’s urine and blood from 30 hours to 4 days after transplant belonged to the donor lineage (Figure 4, A and B). Though we were unable to amplify any recipient virus in any of the samples before and after transplantation, 1 HIV *env* sequence amplified from urine 30 hours after transplant represents a distinct viral lineage that groups separately from all donor and donor-derived sequences (Figure 4, A and B), likely representing the recipient virus. Similarly to the transplant cases discussed above, no donor-derived sequences could be amplified in any of the samples collected during follow-up visits.

**Figure 5:**
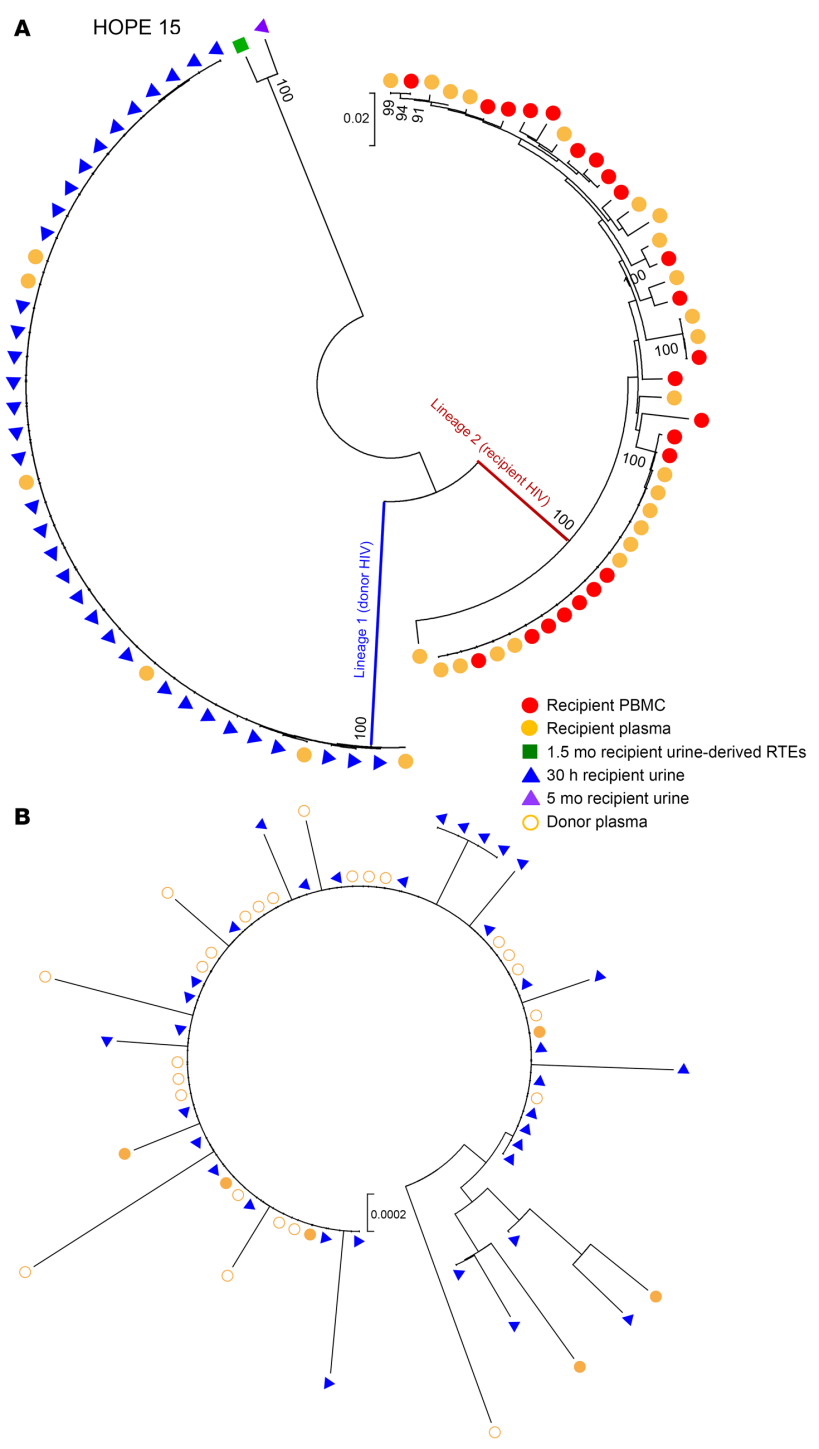
Phylogenetic tree analysis of HIV *env* sequences amplified from Hope 15 recipient before and up to 1 year after kidney transplant. (**A**) Neighbor-joining phylogenetic tree that includes all of the HIV envelope sequences amplified from blood and urine samples obtained from the kidney-transplant recipient with HIV before and up to 1 month after transplantation of a kidney from a donor with HIV. Two separate viral lineages (lineages 1 and 2) were identified in the recipient 30 hours after transplantation in both urine and plasma. (**B**) All the HIV quasispecies in lineage 1 that were amplified from the recipient’s urine (solid blue triangles for cell-free viral RNA) and plasma (solid orange circles) at 30 hours after transplantation are genetically related to the donor virus (open shapes). Only donor plasma and allograft biopsy samples were available for analysis. Bootstrap values over 80% are indicated.

HOPE 15 represents an interesting case where the recipient received a kidney from a donor with a reactive HIV-1/2 nucleic acid test (NAT), but a nonreactive HIV antibody test to groups M and O. In combination with donor clinical epidemiology, this strongly suggests acute HIV infection. In this recipient, we detected a separate viral lineage in both urine and plasma samples collected 30 hours after transplantation (Figure 5A). All the *env* sequences (41 total) amplified at 30 hours after transplant corresponded to this separate viral lineage (Figure 5B) and were almost identical on the nucleotide level (Supplemental Figure 2). Surprisingly, at both 1.5 months and 5 months after transplant, we detected a third HIV strain in the urine-derived renal epithelial cells and urine supernatants, respectively. This third virus was highly dissimilar from both the donor and recipient lineages that had been amplified previously, and recombination analysis using the recombination analysis program (RAPR) (25) determined that these 2 sequences were not recombinants of the 2 viruses (Supplemental Figure 3). Additionally, these 2 sequences were predicted to use CCR5 coreceptors, while all the other HIV sequences amplified in this recipient were predicted to use CXCR4 (CCR5 false-positive rate <10%). Given that the donor was acutely infected, these findings are most likely indicative of HIV-1 infection of the recipient with 2 distinct viruses, with the second virus residing in the kidney. Phylogenetic analyses of the recipients who did not have detectable donor virus after transplantation are shown in Supplemental Figures 4–10.

### Identification of HIV sequences in donor kidney biopsies.

In the 12 transplant cases analyzed, we were able to identify HIV *env* sequences in 6 of the 12 kidney biopsies taken from the donor allografts prior to implantation. Those sequences were identified both in donors who were viremic (HOPE 1) as well as those who had an undetectable or low VL (HOPE 5, 6, 8, 9, and 11). For 3 of the 4 transplant cases in which we were also able to amplify sequences from the donor plasma and PBMCs (HOPE 1, 8, 9), the kidney biopsy sequences clustered separately from the urine and blood-derived sequences (Figure 1B and Supplemental Figures 7 and 8). Because viral DNA and RNA were extracted directly from the flash-frozen kidney biopsy, we cannot determine the exact cell origin of these viruses.

### Low-level viremia in posttransplant samples.

Longitudinal analysis of plasma samples revealed the presence of low-level viremia (between 20 and 200 copies/mL) at multiple time points after transplantation in 4 of the recipients (HOPE 1, 2, 4 and 8). The observed blips are likely due to random biological and statistical variation around mean steady-state viremia from latently infected cells that release virus periodically (26–29). Phylogenetic analysis of HIV *env* sequences amplified from those plasma samples identified several genetically identical sequences across various time points after transplantation in some of the recipients, indicating the presence of expanded clones of HIV-infected cells producing small amounts of virus despite continuous ART. For example, in the HOPE 1 recipient, we observed that those sequences were from a phylogenetically distinct compartment compared with PBMC-derived sequences (Figure 1A). It has been previously shown that in ART-treated patients, more than 98% of viral DNA and RNA persist in lymphoid tissues, including lymph nodes (LNs) and gut-associated lymphoid tissue (30). To determine whether those compartmentalized plasma viruses originated from LNs, we amplified HIV *env* sequences from iliac LN biopsies collected from the transplant recipient at the time of kidney transplantation. Interestingly, all of the HIV *env* sequences amplified from LNs clustered together with the PBMC-derived sequences, suggesting that LNs are not the source of those compartmentalized plasma viruses observed in this recipient (Figure 1A). Similarly, in the HOPE 2 recipient (Supplemental Figure 4), we amplified 28 HIV *env* sequences in plasma at 9 months after transplantation (VL <20 copies/mL) and the majority of them (20/28) were identical to each other and to sequences amplified at earlier time points (Supplemental Figure 10). We amplified HIV *env* sequences from iliac LN biopsies collected also from this recipient at the time of kidney transplantation, and similarly to HOPE 1, LNs did not appear to be the source of those identical plasma sequences (Supplemental Figure 4).

### Graft function and posttransplant comorbidities.

Kidney function was monitored over time following transplantation by assessing creatinine and proteinuria levels in urine. Graft and patient survival were 100% at 1 year. Two transplant recipients (HOPE 6 and 15) experienced proteinuria after transplant with subsequent biopsy notable for recurrence of focal segmental glomerulosclerosis (FGS) possibly due to HIVAN with no evidence of substantial allograft rejection. One of the 12 patients (HOPE 12) died during the follow-up period (23 months after transplant) due to complications from large B-cell lymphoma arising in HHV8-associated multicentric Castleman disease. All 12 patients in the cohort were considered intermediate risk for CMV reactivation (all donors and recipients were CMV IgG positive) and received 6 months of CMV prophylaxis after transplant with valganciclovir per institutional protocol. Two patients (HOPE 1 and 6) developed clinically substantial CMV viremia (CMV DNA PCR >137 IU/ML) within 2 years of transplantation. In both cases, CMV was suppressed with a short course of a treatment dose of valganciclovir. No tissue-invasive disease was noted. Four of the 12 patients (HOPE 6, 9, 11, and 12) developed detectable BK viremia within 2 years of transplant. In 2 cases (HOPE 6 and 9), mycophenolate was temporarily reduced, and the BK viremia resolved. In the other 2 cases (HOPE 11 and 12), mycophenolate was stopped completely due to ongoing BK viremia. Only 1 of these patients, HOPE 12, had evidence of BK nephropathy confirmed by biopsy. A summary of posttransplant complications and allograph function for all 12 kidney transplant recipients is shown in Supplemental Table 2.

## Discussion

The phylogenetic analysis performed on HIV quasispecies isolated before and after 12 HIV-positive–to–HIV-positive kidney transplantations revealed several important findings. We demonstrated that HIV quasispecies harbored in the kidney from donors with HIV can be found in the urine and blood of the recipient just hours after kidney transplantation; this occurs more frequently in recipients who receive an organ from a viremic donor, consistent with the donated kidney as the source of those viruses. Although several donor-derived HIV sequences were readily amplified from plasma and urine up to 16 days after transplantation in some recipients, analysis of follow-up samples collected years after transplantation failed to detect any donor-derived HIV sequence, suggesting that the continuous administration of ART limits and contains the spread of the donor virus in the recipient. These data are consistent with our previously reported case (22) as well as data from other studies investigating HIV superinfection in HIV-positive–to–HIV-positive kidney and liver transplant recipients (9, 11).

Interestingly, even though identification of donor-derived virus in recipient urine and blood samples was largely dependent on the plasma VL of the donor, we were able to amplify HIV *env* sequences from donor kidney biopsies in 6 of the 12 transplant donors, including those without viremia at the time of transplant, and from the renal epithelial cells shed in the urine of 2 recipients soon after transplantation, demonstrating infection of the allograft. Whether those kidney viruses have the potential to reactivate and fuel systemic infection during periods of inadequate ART exposure remains to be determined. However, in the recipient that experienced a viremic episode 3.5 years after transplant, we did not detect donor virus in plasma (urine was not collected), suggesting that the donor virus was not reactivated despite temporary ART withdrawal in this case. Phylogenetic analysis of the rebounding plasma viruses showed very little diversity. Out of the 245 *env* sequences amplified at that time point, 113 were identical on the amino acid level and 45 additional sequences varied by only 1 amino acid. The remaining 87 sequences also showed very little diversity, and though they differed from the master sequence, the majority (61) were identical to each other. These data indicate that these viruses may have come from a clonally expanded cell carrying identical proviruses (31–34).

In line with our previous reports (15, 22, 24), the comparison between urine- and blood-derived sequences from both donors and recipient HIV strains demonstrated compartmentalization of the urine-derived HIV sequences. Despite continuous ART, blips in VL (ranging from 20 to 200 copies/mL) were observed at several time points after transplantation in several recipients. We observed clonal amplification of plasma-derived HIV-1 *env* sequences across multiple time points after transplant in several HOPE recipients, as genetically identical sequences were identified at time points separated by multiple weeks. To further explore the source of these viruses, we analyzed the recipient’s LNs in 2 patients (HOPE 1 and HOPE 2 recipients). However, HIV *env* sequences amplified from iliac LN samples collected from these recipients at the time of transplantation demonstrated that all of the LN-derived HIV sequences were intermixed with PBMC sequences, suggesting that in both recipients, the virus was equilibrated between those sites and that LNs were not the source of those compartmentalized plasma viruses. Our data are consistent with results reported from a previous study on HOPE Act transplant recipients showing comparable sequences in paired PBMC and LN samples (35) and the analysis of samples obtained in clinical studies where individuals underwent analytical treatment interruption (ATI), showing little overlap between viruses isolated from plasma during viral rebound and latent viruses isolated from PBMC and LN samples (36, 37). A previous study conducted on kidney-transplant recipients with HIV demonstrated that a longer duration of observation after transplant revealed small increases in plasma HIV RNA despite ART (38). These recipients might belong to a subset of PLWH with nonsuppressible viremia (NSV) on ART (26–29). A recent study demonstrated that NSV is driven by both viral and host immune factors, including the presence of large, clonally expanded reservoirs of proviruses frequently harboring immune escape mutations, integrated in transcriptionally permissive chromosomal regions, within CD4^+^ T cells primed for survival, and in an environment of muted HIV-specific T cell responses (29).

Seven of the 12 transplant recipients encountered some complications after transplantation, including delayed graft function, tubulitis, interstitial fibrosis, immune-complex mesangiopathic glomerulopathy, and HIVAN recurrence in 2 recipients. These complications did not markedly affect graft function in most cases and are consistent with posttransplant issues reported by previous studies looking at outcomes of kidney transplantation in individuals with HIV (39).

There are a few limitations to our study. Although we performed an in-depth longitudinal phylogenetic analysis of the HIV quasispecies in different compartments, we were limited in the volume of biologic specimens collected specifically for these analyses. Sequencing data could not be generated for all samples due to low HIV proviral loads in some samples. This is expected because most recipients had long-standing viral suppression with ART. Despite these limitations, our data together with previous findings suggest that HIV superinfection might not be a considerable clinical concern in well-monitored, ART-suppressed recipients. Nevertheless, further and continuous monitoring of viral populations in these transplant recipients with HIV is needed to understand the long-term clinical and virological implications of the presence of donor HIV in the transplanted kidney.

## Methods

### Sex as a biological variable.

Our study includes both male and female kidney transplant recipients and donors, with an overrepresentation of males in each group (10/12 for both donors and recipients). Given the rare nature of these procedures and our limited sample size, this study cannot establish sex as a biological variable.

### Specimen collection and processing.

Blood (~15 mL) and urine (20–100 mL) specimens were obtained from both donors and recipients before transplantation and at different time points after transplantation from the recipients. At the time of transplantation, we collected a renal biopsy from all 12 donor kidneys as well as a biopsy from iliac LNs from 2 recipients (HOPE 1 and 2). The LN biopsy was processed to obtain a single cell suspension. The kidney biopsies were snap-frozen for subsequent DNA and RNA extractions. Ethylenediaminetetraacetic acid (EDTA) anticoagulated blood samples were processed to isolate plasma and PBMCs by Ficoll gradient centrifugation at 800*g* for 30 minutes. Urine samples were spun at 400*g* for 10 minutes to separate urine supernatants from urinary cells. Supernatants were then filtered through a 0.45 polyvinylidene difluoride (PVDF) μm filter unit to remove cellular debris. Both urine supernatants and plasma samples were subjected to 2 hours of ultracentrifugation at 128,000*g* to pellet HIV virions. Pelleted viruses were then resuspended in 1× PBS and subjected to RNA extraction. The urinary cells were plated on 0.1% gelatin-coated plates in renal epithelial cell growth medium (Lonza, catalog CC-4127) to isolate and expand a population of adherent renal epithelial cells as previously described (40, 41). Three days after plating, cells that did not adhere to the plate (dying renal cells, urethral cells, and lymphocytes) were pelleted. Medium was changed daily to completely remove all nonadherent cells. Renal cells were expanded in culture for 3 to 5 weeks.

### Viral RNA/DNA extraction and cDNA synthesis.

Viral RNA was extracted from concentrated urine and blood plasma by using the QIAGEN EZ1 Virus Mini Kit, version 2.0 (catalog 955134), and was then subjected to cDNA synthesis as previously described (15). Reactions without reverse transcriptase were included as negative controls. RNA and DNA were extracted from the donor kidney biopsy using the QIAGEN AllPrep DNA/RNA Mini Kit (catalog 80204). Viral DNA was extracted from 5 million PBMCs, urine-derived renal cells cultivated for 3 to 5 weeks, and LN-derived cells (HOPE 1 and 2) by using the QIAamp Mini Kit (catalog 51304) following the manufacturer’s instructions.

### Single genome amplification.

The full-length HIV envelope (*env*) gene (~2500 bp) was amplified from urine, donor kidney biopsy, and plasma-derived cDNA as well as from DNA extracted from PBMCs, donor kidney biopsy, urine-derived renal cells, and LNs by performing single genome amplification as previously described (15). All PCR procedures were carried out under PCR clean-room conditions with procedural safeguards against sample contamination.

### Sequencing and phylogenetic analyses.

The *env* gene amplicons were sequenced via the primer walking method. Individual sequence fragments for each amplicon were assembled and edited using the Sequencher program, version 5.4.1 (Gene Codes). Inspection of individual chromatograms allowed for the identification of amplicons derived from single versus multiple templates. The absence of mixed bases at each nucleotide position throughout the entire *env* gene was taken as evidence of amplification from a single viral RNA/cDNA template. Sequences with premature stop codons were excluded from analysis. All alignments were made using Gene Cutter (hiv.lanl.gov). CCR5 coreceptor utilization of env sequences was determined with Geno2pheno (coreceptor) (https://coreceptor.geno2pheno.org/) using a false-positive rate of 10%. Phylogenetic trees were made with MEGA6 (42). Neighbor-joining trees were constructed under the Kimura 2-parameter mode, and the reliability of topologies was estimated by performing bootstrap analysis with 1,000 replicates. A codon-based test of positive selection comparing the kidney-derived sequences and the PBMC-derived sequences shown in Figure 2B was performed to confirm compartmentalization. This method tests the probability of rejecting the null hypothesis of strict neutrality (dN = dS) in favor of the alternative hypothesis (dN > dS), where dS and dN are the numbers of synonymous and nonsynonymous substitutions per site, respectively. *P* values of less than 0.05 were considered significant at the 5% level. The variance of the difference was computed using the bootstrap method (1,000 replicates). Analyses were conducted using the Nei-Gojobori method (43). All ambiguous positions were removed for each sequence pair (pairwise deletion option). Evolutionary analyses were conducted using MEGA11 (44). Highlighter plots were made using Highlighter (hiv.lanl.gov). Sequences obtained from each donor and recipient were blasted and aligned with the sequences from all the donors and recipients to exclude potential contaminations.

### Study approval.

The research protocol was approved by the Duke University Institutional Review Board (Pro00070449 and Pro0040696), and informed consent was obtained from transplant recipients after the nature and possible consequences of the studies were explained.

### Data and materials availability.

All the sequences produced through this study have been deposited into GenBank accession numbers HOPE4: PQ228602–PQ228835; HOPE5: PQ228836–PQ228871; HOPE1: PQ228872–PQ229512; HOPE6: PQ229513–PQ229541; HOPE8: PQ229542–PQ229620; HOPE9: PQ229621–PQ229789; HOPE11: PQ229790–PQ229831; HOPE13: PQ229832–PQ229870; HOPE14: PQ229871–PQ229921; HOPE15: PQ229922–PQ230036; HOPE2: PQ230037–PQ230426).

## Author contributions

TT and HS contributed equally to formal analysis, investigation and writing — original draft. NA contributed to data curation and writing — review and editing. FG contributed to data curation, methodology, software, and writing — review and editing. MK contributed to conceptualization and writing — review and editing. CW contributed to conceptualization and writing — review and editing. MB contributed to conceptualization, data curation, formal analysis, funding acquisition, investigation, methodology, project administration, resources, supervision, validation, visualization, and writing — review and editing. All authors reviewed and approved the final version of the manuscript.

## Supplementary Material

Supplemental data

ICMJE disclosure forms

## Figures and Tables

**Table 1:**
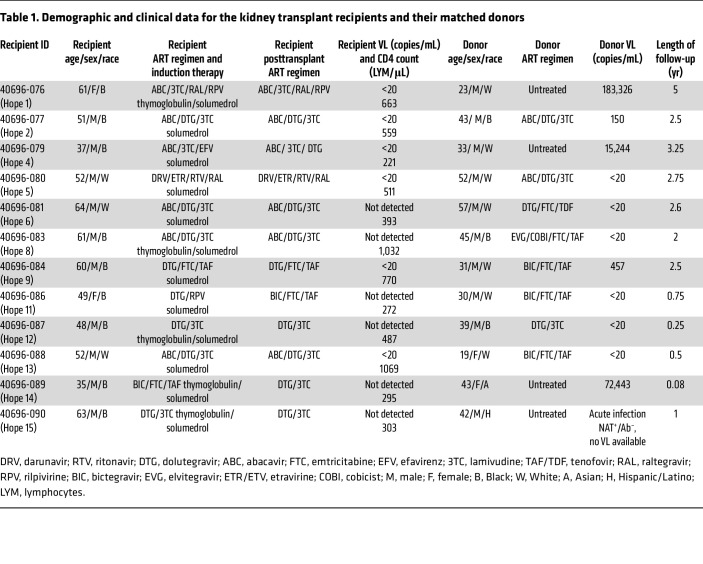
Demographic and clinical data for the kidney transplant recipients and their matched donors

## Version 1 09/10/2024

In-Press Preview

## Version 2 10/15/2024

Electronic publication
