# Supplementary material for: Longitudinal analysis of viral dynamics in HIV+-to-HIV+ HOPE Act kidney-transplant recipients
Source: J Clin Invest. 2024 Sep 10;134(20):e181560. doi: 10.1172/JCI181560 (PMC11473162; doi:10.1172/JCI181560)
Supplement: Supplemental data [file jci-134-181560-s091.pdf]

Supplemental Table 1. Summary of sequence amplification in recipients where donor virus was detected post-transplant.

Patient ID	Timepoint	Sample	Total Number of Sequences	Number of donor-derived Sequences
40696-076 (HOPE 1)	12H	Urine	37	37
		RTE	10	6
		Non-adherent urinary cells	1	
	D3	Plasma	2	2
	D9	Urine	3	1
		RTE	6	
		PBMC	8	
		Plasma	5	
	D16	Urine	1	1
		Non-adherent urinary cells	2	
		Plasma	9	
		PBMC	16	
	D19	PBMC	1	
	D42	Plasma	5	
		PBMC	13	
	D65 (2 mo.)	Plasma	1	
		PBMC	33	
	D107 (3 mo.)	PBMC	27	
	D212 (7 mo.)	Plasma	15	
		PBMC	69	
	D402 (13 mo.)	Plasma	5	
	D510 (16 mo.)	Plasma	1	
	D919 (30 mo.)	Plasma	1	
	D1331 (43 mo.) - ATI	Plasma	245	
40696-079 (HOPE 4)	12H	Urine	23	23
		Plasma	26	4
		PBMC	5	
	D3	Urine	45	45
		Plasma	7	1
	D7	Urine	1	1
	D12	Plasma	2	
	D119 (4 mo.)	PBMC	2	
	D203 (7 mo.)	Plasma	16	
	D355 (12 mo.)	Plasma	2	
		PBMC	57	
40696-086 (HOPE 11)	24H	Plasma	4	2
	D10	PBMC	2	
		RTE	1	
	D40	Plasma	1	
	D271 (9 mo.)	PBMC	2	
40696-089 (HOPE 14)	30H	Urine	13	12
		Plasma	1	1
	D4	Urine	5	5
		Plasma	1	1
40696-090 (HOPE 15)	30H	Urine	35	35
		Plasma	6	6
	D45	PBMC	12	
		RTE	1	
	D143 (5 mo.)	Urine	1	
		Plasma	4	

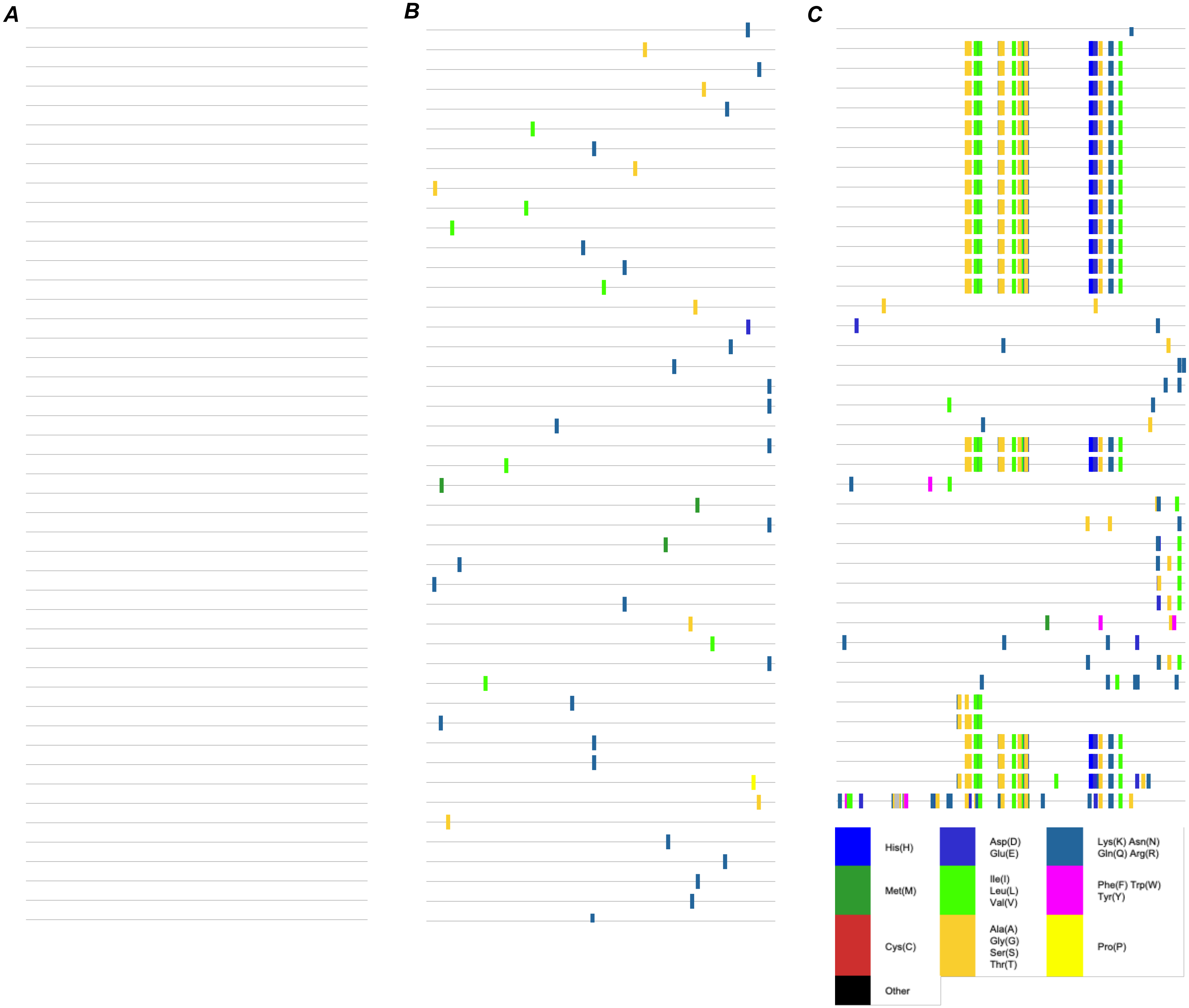
A
B
C
His(H)
Met(M)
Cys(C)
Other
Asp(D) Glu(E)
Ile(I) Leu(L) Val(V)
Ala(A) Gly(G) Ser(S) Thr(T)
Lys(K) Asn(N) Gln(Q) Arg(R)
Phe(F) Trp(W) Tyr(Y)
Pro(P)

Supplemental Figure 1. Highlighter plots of a subset of HIV *env* sequences amplified from HOPE 1 recipient during viremic episode at 3.5 years post kidney transplant. *Panel A* shows a highlighter plot of a subset of the identical HIV envelope sequences (113 total) amplified from the plasma sample obtained from the HIV-positive kidney-transplant recipient during a viremic episode 3.5 years after transplantation. *Panel B* shows a highlighter plot of a subset of HIV envelope sequences with a single amino acid mutation (45 total), and *Panel C* shows a highlighter plot of a subset of HIV envelope sequences with more than one amino acid mutation amplified during the viremic episode. All the sequences (245 total) amplified at this time point belonged to the recipient virus. No donor virus could be amplified in this plasma sample.

HOPE 15

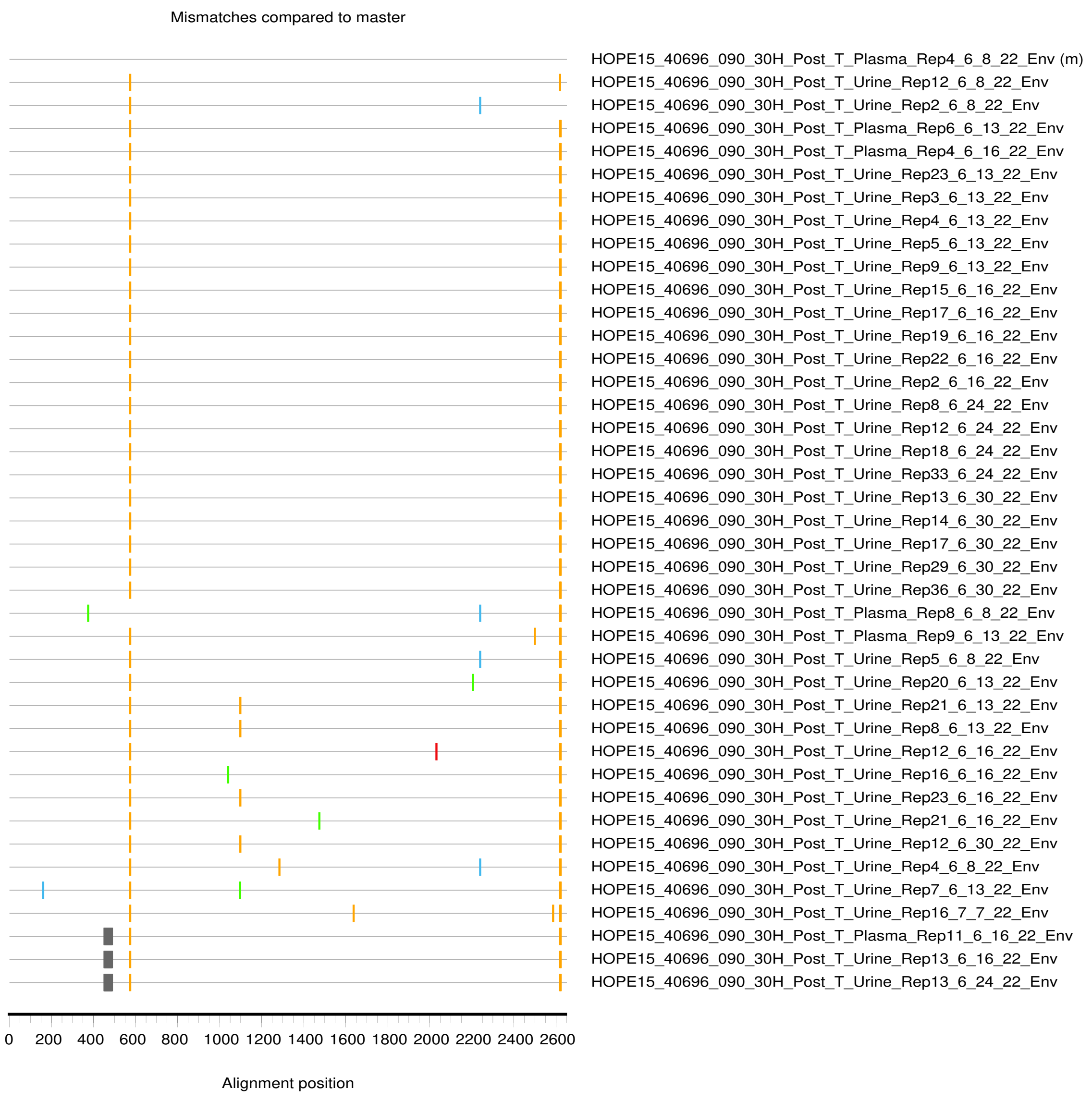

Supplemental Figure 2. Highlighter plot of HIV *env* sequences amplified from HOPE 15 recipient 30 hours post-transplant. All the env sequences (41 total) amplified at 30 hours post-transplant from both urine and plasma corresponded to the donor HIV and were almost identical on the nucleotide level.

HOPE 15

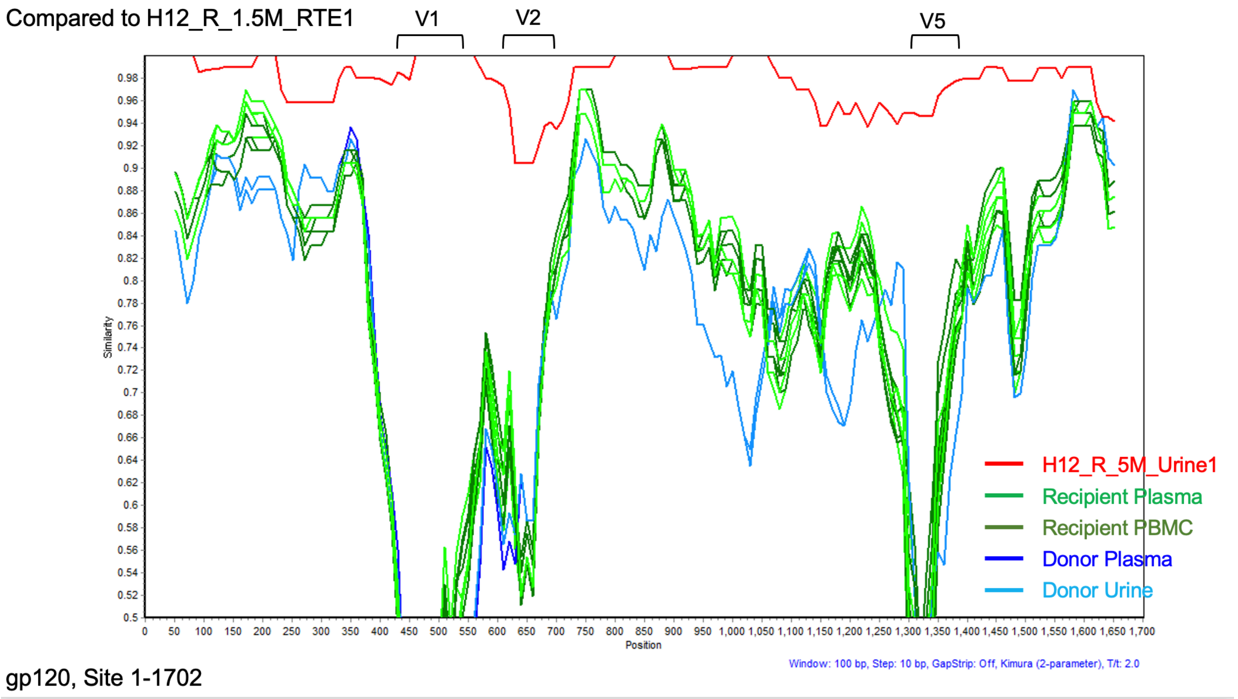

Supplemental Figure 3. Recombination analysis of HOPE 15 sequences detected in urine at 1.5 and 5 months post-transplant using the Recombination Analysis PRogram (RAPR). Shown is a Simplot graph comparing the gp120 portion of the HIV Env sequences from the different sources shown in the figure legend. The plot shows that the two sequences from the third lineage detected in urine-derived renal tubule epithelial cells (RTE) and urine at 1.5 and 5 months post-transplant respectively, are very different from both recipient and donor viruses, therefore they are not the result of recombination between the recipient and donor strains. Higher sequence diversity from both recipient and donor viruses were observed at the V1V2 and V3 regions of gp120.

HOPE 2

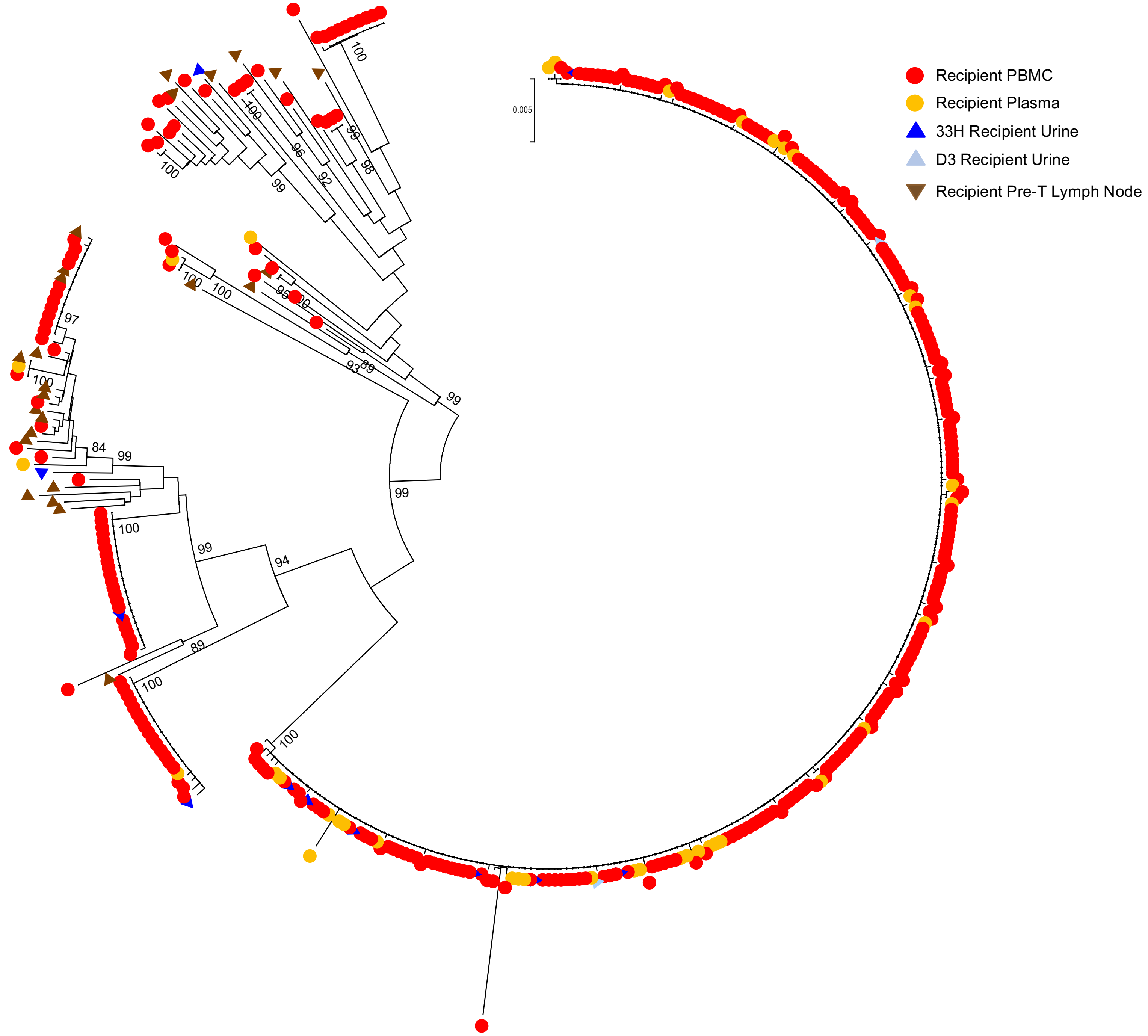
Recipient PBMC
Recipient Plasma
33H Recipient Urine
D3 Recipient Urine
Recipient Pre-T Lymph Node
0.005
100
99
98
96
92
99
100
100
97
100
84
99
100
99
94
89
100
99
100
100
99
93
100

Supplemental Figure 4. Phylogenetic tree analysis of HIV *env* sequences amplified from HOPE 2 recipient before and up to 2.5 years post kidney transplant. Shown is a neighbor-joining phylogenetic tree that includes all of the HIV envelope sequences amplified from blood, urine and lymph node samples obtained from the HIV-positive kidney-transplant recipient before and up to 2.5 years after transplantation of a kidney from an HIV-positive donor. No donor virus could be amplified in this recipient. Bootstrap values over 80% are indicated. All the urine-derived HIV sequences (11/11) were predicted to use CCR5 co-receptors (CCR5 false-positive rate < 10%).

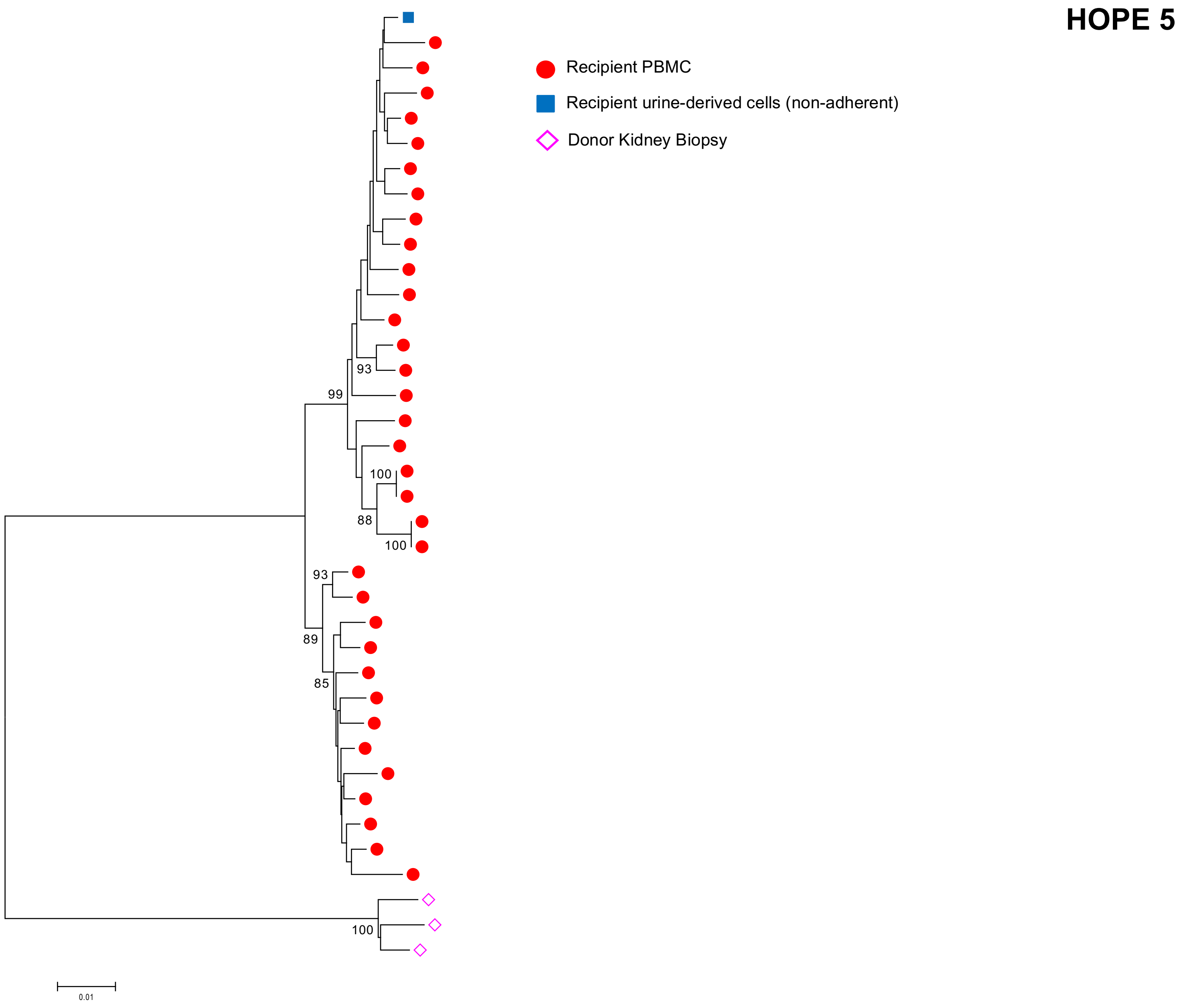
HOPE 5
Recipient PBMC
Recipient urine-derived cells (non-adherent)
Donor Kidney Biopsy
93
99
100
88
100
93
89
85
100
0.01

Supplemental Figure 5. Phylogenetic tree analysis of HIV *env* sequences amplified from Hope 5 recipient before and up to 2.75 years post kidney transplant. Shown is a neighbor-joining phylogenetic tree that includes all of the HIV envelope sequences amplified from blood and urine samples obtained from the HIV-positive kidney-transplant recipient before and up to 2.75 years after transplantation of a kidney from an HIV-positive donor. No donor virus could be amplified in this recipient. However, several HIV env sequences were amplified from the kidney biopsy taken from the allograft before implantation. Bootstrap values over 80% are indicated. All the kidney-derived HIV sequences (3/3) and 13 of the PBMC-derived HIV sequences (13/34) were predicted to use CCR5 co-receptors (CCR5 false-positive rate < 10%).

HOPE 6

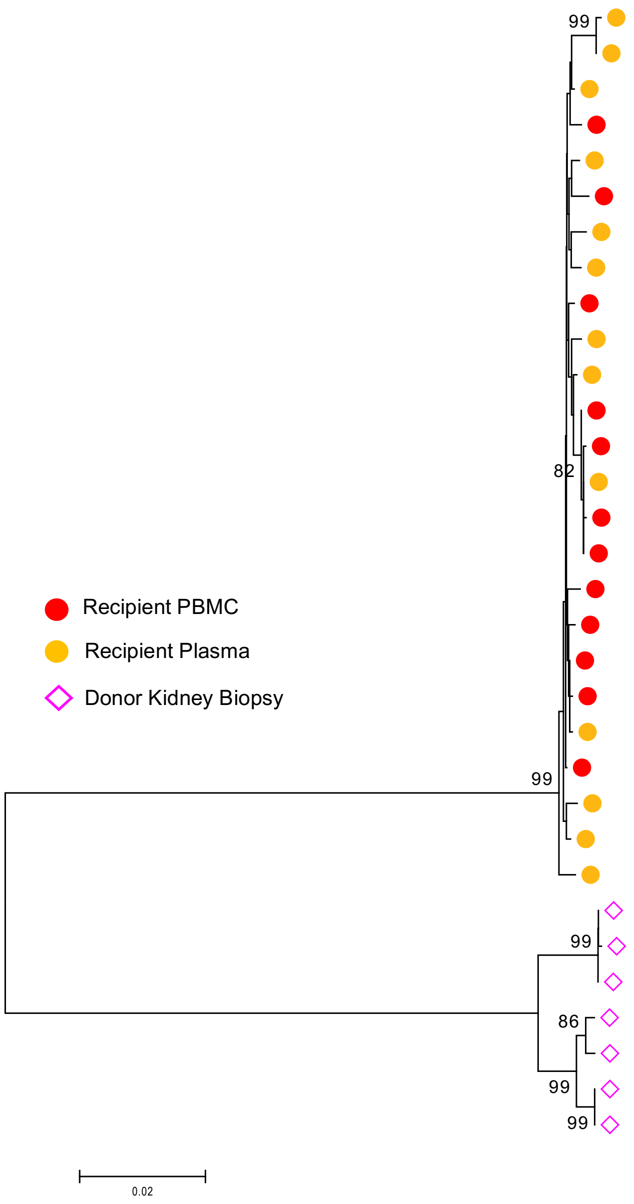

Supplemental Figure 6. Phylogenetic tree analysis of HIV *env* sequences amplified from HOPE 6 recipient before and up to 2.6 years post kidney transplant. Shown is a neighbor-joining phylogenetic tree that includes all of the HIV envelope sequences amplified from blood samples obtained from the HIV-positive kidney-transplant recipient before and up to 2.6 years after transplantation of a

kidney from an HIV-positive donor. No donor virus could be amplified in this recipient. However, several HIV env sequences were amplified from the kidney biopsy taken from the allograft before implantation. Bootstrap values over 80% are indicated. All the kidney- (7/7) and PBMC-derived HIV sequences (12/12) and 12 of the 13 plasma-derived HIV sequences were predicted to use CCR5 co-receptors (CCR5 false-positive rate < 10%).

HOPE 8

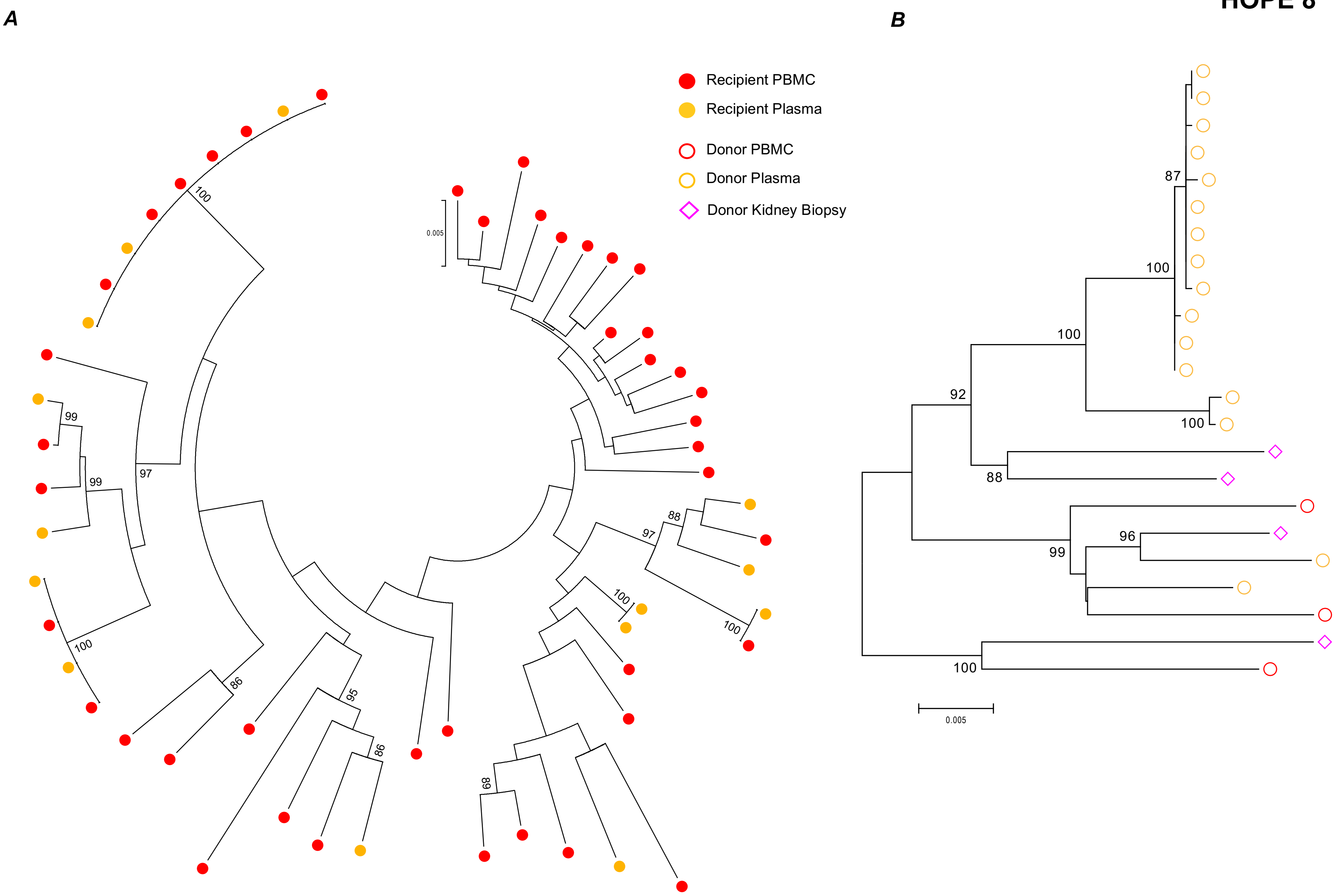

A
B
Recipient PBMC
Recipient Plasma
Donor PBMC
Donor Plasma
Donor Kidney Biopsy
0.005
100
99
99
97
100
86
95
86
89
100
100
97
88
0.005
87
100
100
92
100
88
96
99
100

Supplemental Figure 7. Phylogenetic tree analysis of HIV *env* sequences amplified from Hope 8 recipient before and up to 2 years post kidney transplant. *Panel A* shows a neighbor-joining phylogenetic tree that includes all of the HIV envelope sequences amplified from blood samples obtained from the HIV-positive kidney-transplant recipient before and up to 2 years after transplantation of a kidney from an HIV-positive donor. No donor virus could be amplified in this recipient. However, several HIV env sequences were amplified from the kidney biopsy (open pink diamonds) taken from the allograft before implantation (*panel B*). Bootstrap values over 80% are indicated. All the donor-derived HIV sequences (23/23) were predicted to use CCR5 co-receptors, while all the recipient-derived HIV sequences (57/57) were predicted to use CXCR4 (CCR5 false-positive rate < 10%).

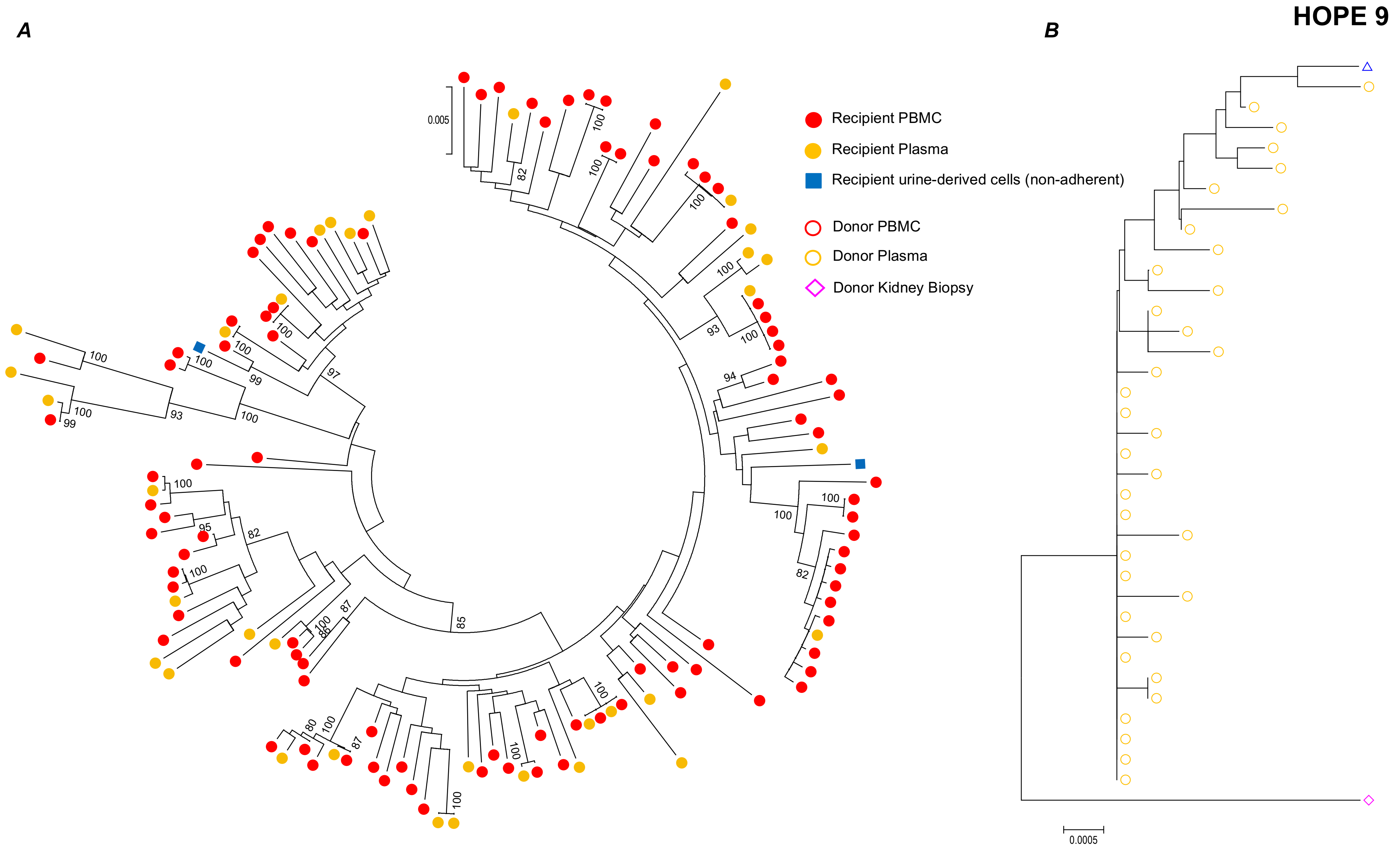

Supplemental Figure 8. Phylogenetic tree analysis of HIV *env* sequences amplified from Hope 9 recipient before and up to 2.5 years post kidney transplant. *Panel A* shows a neighbor-joining phylogenetic tree that includes all of the HIV envelope sequences amplified from blood samples obtained from the HIV-positive kidney-transplant recipient before and up to 2.5 years after transplantation

of a kidney from an HIV-positive donor. No donor virus could be amplified in this recipient. However, a HIV env sequence was amplified from the kidney biopsy (open pink diamond) taken from the allograft before implantation (*panel B*). Bootstrap values over 80% are indicated. All the donor- and recipient-derived HIV sequences were predicted to use CCR5 co-receptors (CCR5 false-positive rate < 10%).

HOPE 13

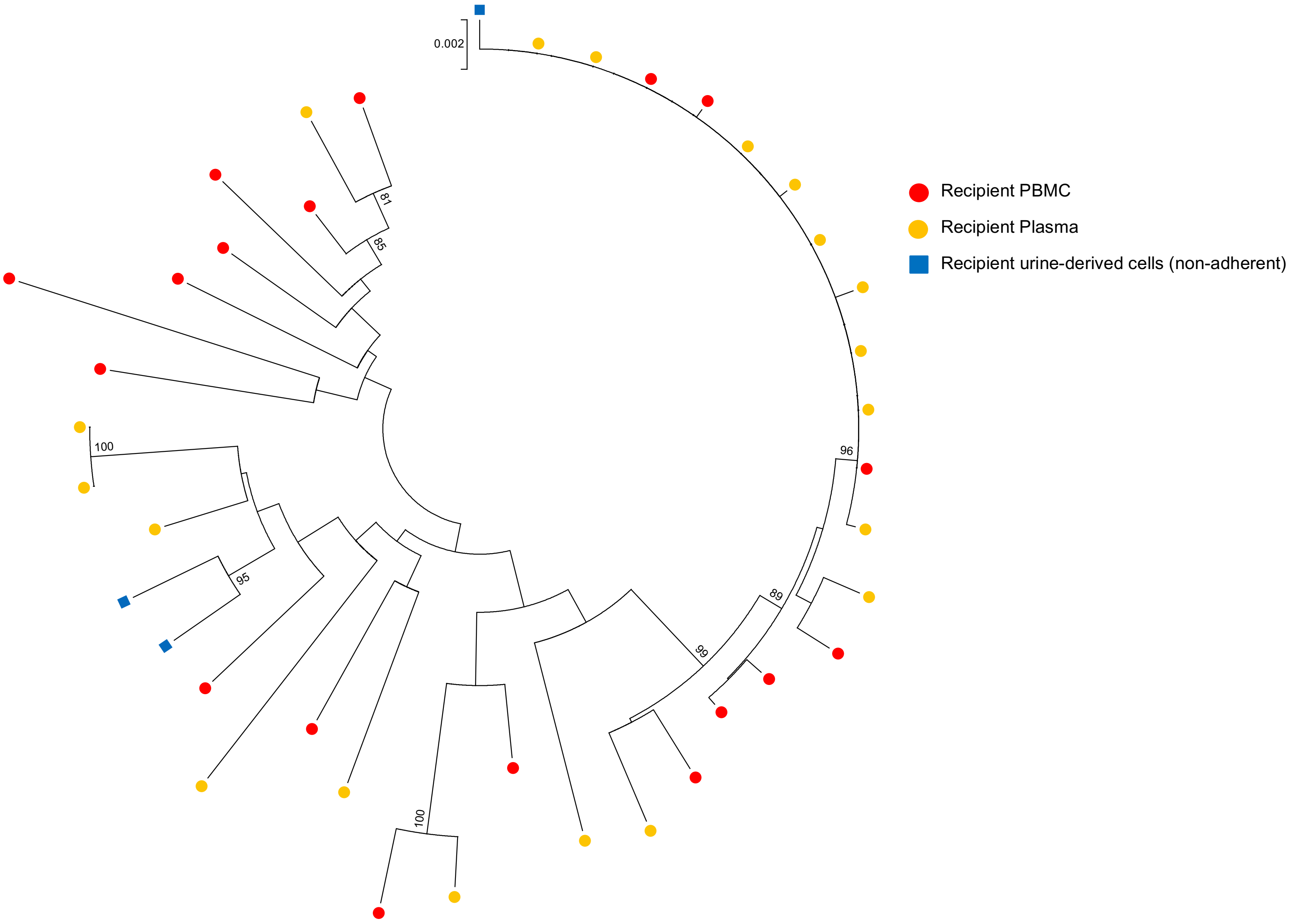

Supplemental Figure 9. Phylogenetic tree analysis of HIV *env* sequences amplified from HOPE 13 recipient before and up 6 months post kidney transplant. Shown is a neighbor-joining phylogenetic tree that includes all of the HIV envelope sequences amplified from blood and urine samples obtained from the HIV-positive kidney-transplant recipient before and up to 6 months after

transplantation of a kidney from an HIV-positive donor. No donor virus could be amplified in this recipient. Bootstrap values over 80% are indicated. All the HIV sequences for this recipient were predicted to use CCR5 co-receptors (CCR5 false-positive rate < 10%).

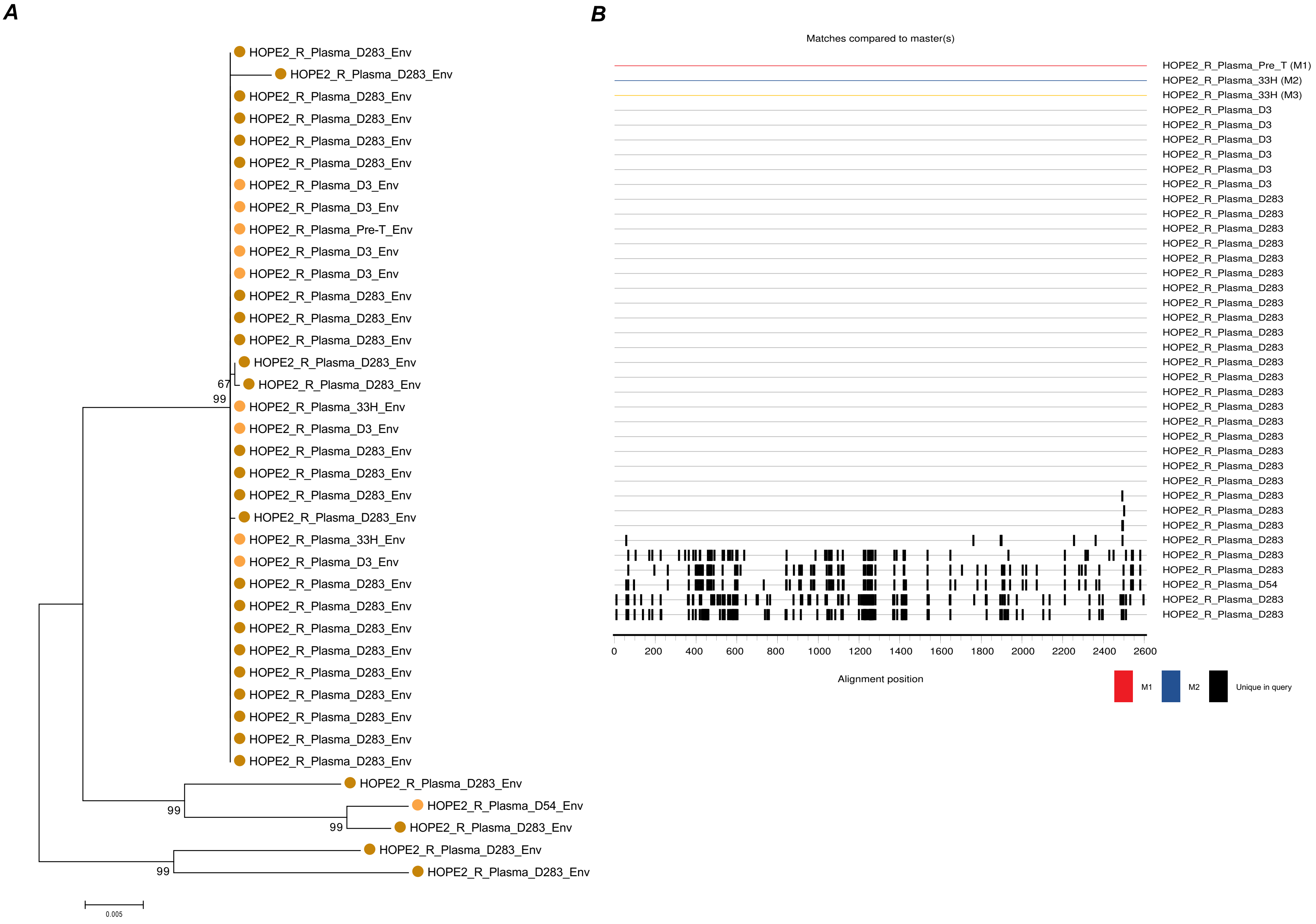
A
HOPE2_R_Plasma_D283_Env
HOPE2_R_Plasma_D283_Env
HOPE2_R_Plasma_D283_Env
HOPE2_R_Plasma_D283_Env
HOPE2_R_Plasma_D283_Env
HOPE2_R_Plasma_D283_Env
HOPE2_R_Plasma_D3_Env
HOPE2_R_Plasma_D3_Env
HOPE2_R_Plasma_Pre-T_Env
HOPE2_R_Plasma_D3_Env
HOPE2_R_Plasma_D3_Env
HOPE2_R_Plasma_D283_Env
HOPE2_R_Plasma_D283_Env
HOPE2_R_Plasma_D283_Env
HOPE2_R_Plasma_D283_Env
67
HOPE2_R_Plasma_D283_Env
99
HOPE2_R_Plasma_33H_Env
HOPE2_R_Plasma_D3_Env
HOPE2_R_Plasma_D283_Env
HOPE2_R_Plasma_D283_Env
HOPE2_R_Plasma_D283_Env
HOPE2_R_Plasma_D283_Env
HOPE2_R_Plasma_33H_Env
HOPE2_R_Plasma_D3_Env
HOPE2_R_Plasma_D283_Env
HOPE2_R_Plasma_D283_Env
HOPE2_R_Plasma_D283_Env
HOPE2_R_Plasma_D283_Env
HOPE2_R_Plasma_D283_Env
HOPE2_R_Plasma_D283_Env
HOPE2_R_Plasma_D283_Env
HOPE2_R_Plasma_D283_Env
HOPE2_R_Plasma_D283_Env
HOPE2_R_Plasma_D283_Env
99
HOPE2_R_Plasma_D54_Env
99
HOPE2_R_Plasma_D283_Env
HOPE2_R_Plasma_D283_Env
99
HOPE2_R_Plasma_D283_Env
0.005
B
Matches compared to master(s)
HOPE2_R_Plasma_Pre_T (M1)
HOPE2_R_Plasma_33H (M2)
HOPE2_R_Plasma_33H (M3)
HOPE2_R_Plasma_D3
HOPE2_R_Plasma_D3
HOPE2_R_Plasma_D3
HOPE2_R_Plasma_D3
HOPE2_R_Plasma_D3
HOPE2_R_Plasma_D3
HOPE2_R_Plasma_D283
HOPE2_R_Plasma_D283
HOPE2_R_Plasma_D283
HOPE2_R_Plasma_D283
HOPE2_R_Plasma_D283
HOPE2_R_Plasma_D283
HOPE2_R_Plasma_D283
HOPE2_R_Plasma_D283
HOPE2_R_Plasma_D283
HOPE2_R_Plasma_D283
HOPE2_R_Plasma_D283
HOPE2_R_Plasma_D283
HOPE2_R_Plasma_D283
HOPE2_R_Plasma_D283
HOPE2_R_Plasma_D283
HOPE2_R_Plasma_D283
HOPE2_R_Plasma_D283
HOPE2_R_Plasma_D283
HOPE2_R_Plasma_D283
HOPE2_R_Plasma_D283
HOPE2_R_Plasma_D283
HOPE2_R_Plasma_D283
HOPE2_R_Plasma_D283
HOPE2_R_Plasma_D283
HOPE2_R_Plasma_D283
HOPE2_R_Plasma_D283
HOPE2_R_Plasma_D54
HOPE2_R_Plasma_D283
HOPE2_R_Plasma_D283
0
200
400
600
800
1000
1200
1400
1600
1800
2000
2200
2400
2600
Alignment position
M1
M2
Unique in query

Supplemental Figure 10. Phylogenetic tree analysis of HIV *env* sequences amplified from plasma samples of Hope 2 recipient at the indicated time points post kidney transplant. *Panel A* shows is a neighbor-joining phylogenetic tree that includes HIV envelope sequences amplified from plasma samples obtained from the HIV-positive kidney-transplant recipient before and up to 9 months (D283) after transplantation of a kidney from an HIV-positive donor. *Panel B* shows a highlighter plot demonstrating that the majority of plasma sequences amplified at 9 months post-transplant are identical to each other and to sequences amplified at earlier time points post-transplant.

Supplemental Table 2. Post-Transplant Complications and Allograft Function

Recipient ID	Induction therapy	Maintenance immunosuppression	Allograft dysfunction or rejection	Post-Transplant CKD*
40696-076 (Hope 1)	Thymoglobulin/solumedrol	Prednisone, tacrolimus, mycophenolate	None	No
40696-077 (Hope 2)	Solumedrol	Prednisone, tacrolimus, mycophenolate	None	No
40696-079 (Hope 4)	Solumedrol	Prednisone, tacrolimus, mycophenolate	Biopsy proven AMR and borderline ACR with minimal tubulitis. Improved creatinine after high dose solumedrol.	Yes
40696-080 (Hope 5)	Solumedrol	Prednisone, tacrolimus, mycophenolate	Slow graft function initially with early Banff 2A rejection treated with thymoglobulin followed by AKI with borderline T cell rejection and inflammation attributed to calcineurin inhibitor	Yes
40696-081 (Hope 6)	Solumedrol	Prednisone, tacrolimus, mycophenolate	Delayed graft function with initial recovery. Late development of proteinuria with subsequent biopsy notable for FSGS and with severe arteriolar hyalinosis possibly due to HIVAN. Concern for contribution of toxicity from mTOR kinase inhibitor and tenofovir led to switch from sirolimus to tacrolimus and from BIC/FTC/TAF to ABC/DTG/3TC. CKD with slowly rising creatinine	Yes
40696-083 (Hope 8)	Thymoglobulin/solumedrol	Prednisone, tacrolimus, mycophenolate	None	Yes
40696-084 (Hope 9)	Solumedrol	Prednisone, tacrolimus, mycophenolate	None	No
40696-086 (Hope 11)	Solumedrol	Prednisone, tacrolimus, mycophenolate	None	No
40696-087 (Hope 12)	Thymoglobulin/solumedrol	Prednisone, tacrolimus, mycophenolate	Kidney biopsy with lyphoplasmacytic inflammation and interstitial fibrosis and tubular atrophy in the setting of post-transplant transplant Large B-cell lymphoma arising in HHV8-associated multicentric Castleman disease leading to renal failure requiring initiation of renal replacement therapy eventual Castleman disease associated death 23 months post-transplant	Yes
40696-088 (Hope 13)	Solumedrol	Prednisone, tacrolimus, mycophenolate	Late immune-complex mesangiopathic glomerulopathy with deposition of C1q requiring augmentation of immunosuppression with monthly belatacept and return of good renal function	No
40696-089 (Hope 14)	Thymoglobulin/solumedrol	Prednisone, tacrolimus, mycophenolate	Delayed graft function in the context of ureteral implantation into the peritoneum (anastomotic leak) status post repair with tubular and vascular injury related to tacrolimus and/or tenofovir.	Yes
40696-090 (Hope 15)	Thymoglobulin/solumedrol	Prednisone, tacrolimus, mycophenolate	Complications with early proteinuria within 3-4 months post-transplant. Subsequent biopsy notable for potential for early recurrent FSGS possibly due to HIVAN. Antiretroviral regimen was augmented with addition of doravirine to dolutegravir-lamivudine.	No

CKD, chronic kidney disease is defined as the persistence of the damage or decreased function present for at least three months at the end of the follow up period, in line with KDOQI and KDIGO guidelines
FSGS, Focal segmental glomerulosclerosis
